# NbTMP14 Is Involved in Tomato Spotted Wilt Virus Infection and Symptom Development by Interaction with the Viral NSm Protein

**DOI:** 10.3390/v13030427

**Published:** 2021-03-07

**Authors:** Jin Zhan, Huiping Shi, Weimin Li, Chao Zhang, Yongqiang Zhang

**Affiliations:** Biotechnology Research Institute, Chinese Academy of Agricultural Sciences, Beijing 100081, China; jin.zhan@allwegene.com (J.Z.); shihuiping2021@163.com (H.S.); liweimin01@caas.cn (W.L.)

**Keywords:** tomato spotted wilt virus, NSm, TMP14, molecular mechanism, symptoms

## Abstract

Tomato spotted wilt virus (TSWV) is one of the most destructive plant viruses, causing severe losses in many important crops worldwide. The non-structural protein NSm of TSWV is a viral movement protein that induces viral symptoms. However, the molecular mechanisms by which NSm contributes to symptom development are unclear. Here, we present evidence that NSm directly interacts with *Nicotiana benthamiana* chloroplast thylakoid membrane protein TMP14 (NbTMP14) by yeast two-hybrid and bimolecular fluorescence complementation (BiFC) assays. The interaction between NSm and NbTMP14 led to the translocation of the NbTMP14 protein from the chloroplast to the cytoplasm in TSWV-infected plants, and overexpressing NSm decreased NbTMP14 mRNA accumulation. In addition, abnormal chloroplasts and starch accumulation were observed in TSWV-infected plants. Silencing of NbTMP14 by TRV VIGS also showed similar results to those of TSWV-infected plants. Overexpressing NbTMP14 in transgenic *N. benthamiana* plants impeded TSWV infection, and silencing NbTMP14 in *N. benthamiana* plants increased disease symptom severity and virus accumulation. To our knowledge, this is the first report showing that the plant chloroplast TMP14 protein is involved in viral infection. Knowledge of the interaction between NSm and NbTMP14 advances our understanding of the molecular mechanisms underlying TSWV symptom development and infection.

## 1. Introduction

Tomato spotted wilt virus (TSWV), a type member of the order *Bunyavirales*, family *Tospoviridae* and genus *Orthotospovirus*, is one of the most devastating plant viruses worldwide, causing severe economic losses to many important agronomic crops [1,2,3,4,5]. TSWV infects more than one thousand plant species from 84 different families, with typical symptoms including chlorosis, necrosis, ring spots, stunting, and ring/line patterns affecting leaves, stems, and fruits [6,7,8,9]. TSWV is an enveloped negative-strand RNA virus with three genomic RNAs denoted L, M, and S. The L RNA segment has negative polarity and contains one large open reading frame (ORF) that encodes a viral RNA-dependent RNA polymerase (RdRp). The M segment has ambisense polarity and encodes two glycoproteins (Gn and Gc) and a non-structural protein (NSm). The S segment is also ambisense and encodes the nucleocapsid (N) protein and a second non-structural protein (NSs) [8,10].

The non-structural protein NSm is a multifunctional protein and plays an important role during virus infection [8,10]. NSm has typical characteristics of plant viral movement proteins, including RNA-binding activity and interaction with a nucleocapsid protein [11], being physically associated with cellular membranes, localization into ER and plasmodesmata (PD) [12,13], PD modification, and tubule formation [14,15,16]. NSm has been identified as the Avr determinant during the Sw-5b-mediated hypersensitive response (HR) [17,18,19]. In addition to these functions, the TMV-based expression system also showed that NSm induced TSWV-like symptoms in *N. benthamiana*; constitutive expression of NSm in transgenic *Nicotiana tabacum* is sufficient to induce severe, infection-like symptoms as well [16,20,21,22]. However, how TSWV NSm modulates disease symptom development remains largely unknown.

Chloroplasts have been implicated as a common target of plant viruses for a long time [23,24,25,26]. The most common viral symptom is leaf chlorosis, reflecting altered pigmentation and structural changes in chloroplasts. The viral influence on chloroplast structure and function usually leads to depleted photosynthetic activity [27,28,29,30]. The 14 kDa thylakoid membrane phosphoprotein (TMP14) is a novel subunit of plant photosystem I (PS I). A previous study demonstrated that Arabidopsis TMP14 is exclusively associated with PS I and co-migrates with PS I-L. Homologues of TMP14 were predicted to exist both in higher plants and cyanobacteria [31]; however, the function of this protein is largely unknown.

In this study, we identified a *N. benthamiana* chloroplast thylakoid membrane protein TMP14 (denoted as NbTMP14) that interacts with TSWV NSm in both yeast and *N. benthamiana* cells. In addition, we also provide evidence to demonstrate that the interaction between TSWV NSm and NbTMP14 could enhance TSWV infection and viral-induced symptoms.

## 2. Materials and Methods

### 2.1. Plant Growth and Virus Inoculation

*N. benthamiana* plants were grown in growth chambers (Model RXZ500D, Jiangnan Motor Factory, Ningbo, China) with a 16-/8-h photoperiod at 25 °C and 60% humidity. Six- to eight-week-old plants of *N. benthamiana* were used for all transient expression analyses and virus inoculations. The TSWV-YN isolate was maintained in *N. benthamiana* plants, and 1 g of systemically infected leaves were macerated in 5 mL inoculation buffer (50 mM KH_2_PO4, pH 7.0, 1% Celite). Samples were then diluted 100 times for the experiments of resistance identification. Virus-infected plants and/or agroinfiltrated leaves were maintained under the same conditions [32].

### 2.2. Plasmid Construction and N. benthamiana Transformation

The full-length sequences of NSm and NbTMP14 (Sol Genomic Network: Niben101Scf02026g02002.1) were obtained from total RNA isolated from TSWV YN-infected *N. benthamiana* by RT-PCR. The putative signal peptide (SP) of NbTMP14 was predicted using the online software SignalP 4.1 (http://www.cbs.dtu.dk/services/SignalP, accessed on 11 June 2018). All plasmids were constructed using the enzymatic digestion method as detailed previously [21]. All the constructs were sequenced before use; information about the sequences of all the primers and plasmids is provided in Table S1.

The overexpression vector was introduced into *N. benthamiana* plants by *A. tumefaciens* (strain LBA4404)-mediated transformation as described previously [28] with modifications. All *Agrobacteria* were grown overnight to an OD_600_ = 0.6–1.0, pelleted, resuspended in MS culture medium, and adjusted to an OD_600_ = 0.2. Aseptic *N. benthamiana* leaves were cut into small pieces, cultured and shaken for 10 min. Bacteria on the leaves were drained with aseptic filter paper, and the leaves were placed on MS culture medium at 25 °C in the dark for 3–4 days. After 3–4 days, the leaves were moved from MS culture medium to screening culture medium with a 16-/8-h photoperiod at 25 °C. After 3–4 weeks, the leaves produced buds. When these buds grew to 1–2 cm, they were cut off and transferred to the rooting culture medium. When these roots grew to 1–3 cm, water was added to the medium. Two days later, the medium was washed from the roots, and the roots were transferred to soil.

### 2.3. Y2H and BiFC Assays

For yeast two-hybrid (Y2H) assays, the coding sequences of NbTMP14 and Nsm were cloned into yeast vectors pPR3-N and pBT3-STE to generate pPR3-N-NbTMP14 and pBT3-STE-NSm, respectively. Both pPR3-N-NbTMP14 and pBT3-STE-NSm were co-transformed into yeast strain NMY51, according to a modified yeast transformation protocol [33]. Transformants were plated on minimal synthetic defined (SD)-glucose medium containing 6 mM 3-AT and lacking Trp, Leu and His [34].

The coding sequences of NbTMP14 and Nsm were cloned into BiFC vectors, pYCE and pYNE, to generate pYCE-NbTMP14 and pYNE-NSm, respectively. All *Agrobacterium* were grown overnight to an OD_600_ = 0.6–1.0, pelleted, and resuspended in infiltration buffer MMA (10 mM MgCl_2_, 10 mM 2-Morpholinoethanesulfonic acid, and 200 μM acetosyringone). All Agrobacterium cultures were adjusted to an OD_600_ = 0.6 and induced at room temperature for 2 h; 2YC- NbTMP14 was mixed with 2YN-NSm (1:1). These cultures were infiltrated into *N. benthamiana* leaves grown at a 16-/8-h photoperiod at 25 °C. After 36–48 h, infiltrated tobacco leaves were observed using a confocal laser scanning microscope (LSM700; Zeiss, Munich, Germany) at the following excitation wavelengths: eYFP at 488 nm [34].

### 2.4. Confocal and Electron Microscopy

Subcellular location images were captured with a confocal laser scanning microscope (LSM700; Zeiss) at the following excitation wavelengths: GFP at 488 nm [13].

For transmission electron microscopy (TEM) observation, 1 mm sections of *N. benthamiana* leaves were cut and fixed in 2% (*v*/*v*) glutaraldehyde (Sigma-Aldrich, St. Louis, MI, USA) and 1% osmium tetroxide solution sequentially. After dehydration through a gradient of ethanol, the samples were dried, the ethanol was replaced with isoamyl acetate, and the samples were sprayed with gold particles and observed by TEM (Hitachi-7700, Tokyo, Japan) [35].

### 2.5. TRV-VIGS in N. benthamiana

All *Agrobacterium* were grown overnight to an OD_600_ = 0.6–1.0, pelleted, and resuspended in infiltration buffer MMA containing 10 mM MgCl_2_, 10 mM 2-Morpholinoethanesulfonic acid, and 200 μM acetosyringone. All *Agrobacterium* cultures were adjusted to an OD600 = 0.6, induced at room temperature for 3 h, and pTRV1 was mixed with pTRV2 -GFP or pTRV2-TMP14 (1:1). The cultures were infiltrated into *N. benthamiana* leaves grown with a 16-/8-h photoperiod at 25 °C [36]. 

### 2.6. RT-qPCR and Western Blot Assay

Total RNA was extracted from *N. benthamiana* leaves using TRIzol reagent (Invitrogen, New York, NY, USA). After removal of genomic DNA with RNase-free DNase Ι (TaKaRa), first-strand cDNA was synthesized using the Primerscript RT reagent kit with gDNA Eraser (TaKaRa, Kusatsu, Japan). Quantitative real-time (qRT)-PCR was performed using a 2 × SYBR Premix Ex TaqTM (TaKaRa), and actin was used as the internal control. All qRT-PCR experiments were completed in triplicate using three independent samples [34].

*N. benthamiana* leaves were ground in protein extraction buffer (6 M urea, 1 mM EDTA, 50 mM Tris–HCl, 1% SDS, pH 7.5). Proteins were separated on 10% *w*/*v* SDS-PAA (Sodium dodecylsulfate (SDS)-polyacrylamide (PAA)) gel and transferred to polyvinylidene fluoride by semi-dry blotting and were detected with an antibody (Roche, Basel, Switzerland) [34].

## 3. Results

### 3.1. TSWV NSm Interacted with the N. benthamiana TMP14 Protein

**Figure 1:**
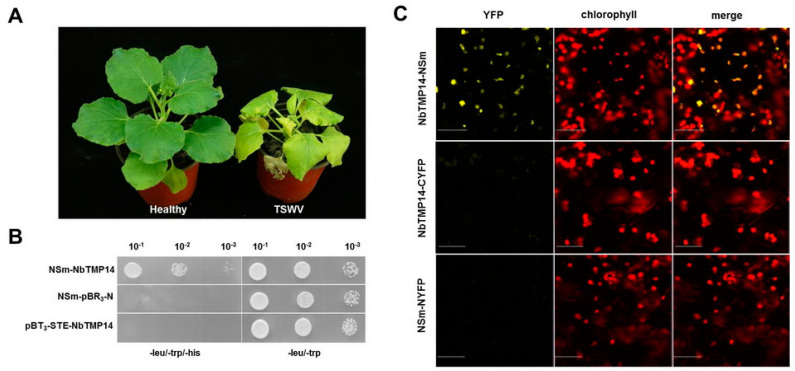
Identification of tomato spotted wilt virus (TSWV) NSm interacting with TMP14 protein in *Nicotiana benthamiana*. (**A**) Symptoms of TSWV-infected *N. benthamiana*, 15 dpi. (**B**) Verification of NbTMP14 interacting with NSm though the yeast two-hybrid system. (**C**) Interaction between NbTMP14 and NSm was investigated by BiFC in leaves of *N. benthamiana*. Scale bars represent 100 µm.

Chlorosis, necrosis, and dwarfing, typical symptoms caused by TSWV infection on *N. benthamiana*, were observed (Figure 1A). The NSm protein is a pathogenic factor of the virus. To identify host factors that interact with NSm during TSWV infection, we screened a *N. benthamiana* cDNA library using TSWV NSm as bait through Y2H assay. One of the positive colonies contained an intact ORF that encoded a thylakoid membrane protein, which shared a high degree of identity with TMP14 in *A. thaliana*; therefore, we denoted it as NbTMP14. A positive interaction between NbTMP14 and NSm was further confirmed though a Y2H system (Figure 1B).

The interaction between NbTMP14 and NSm was further investigated by BiFC in the leaves of *N. benthamiana*. Pairwise expression of pYCE-NbTMP14 and pYNE-NSm by agroinfiltration resulted in a clear yellow fluorescence signal in the cytoplasm of agroinfiltrated cells at 36 h post infiltration (hpi) (Figure 1C). Two other combinations of constructs, pYCE-NbTMP14 and pYNE and pYNE-NSm and pYCE, were injected as negative controls, and no YFP signal was detected. These results demonstrated that NbTMP14 specifically interacts with NSm in both yeast and plant cells.

### 3.2. Sequence Analysis, Expression Pattern, and Subcellular Localization of NbTMP14

In *A. thaliana*, TMP14 is a subunit of PS I in chloroplasts, but there is no evidence to show that it is associated with viral infection. To better understand the biological function and potential role of NbTMP14 during TSWV infection, the sequence and biological features were analyzed. NbTMP14 cDNA encodes a protein containing 182 amino acids, and signal peptide prediction revealed the presence of a chloroplast localized signal peptide (residues 1–20) in its N terminal, in addition to a mature protein (residues 21–182).

**Figure 2:**
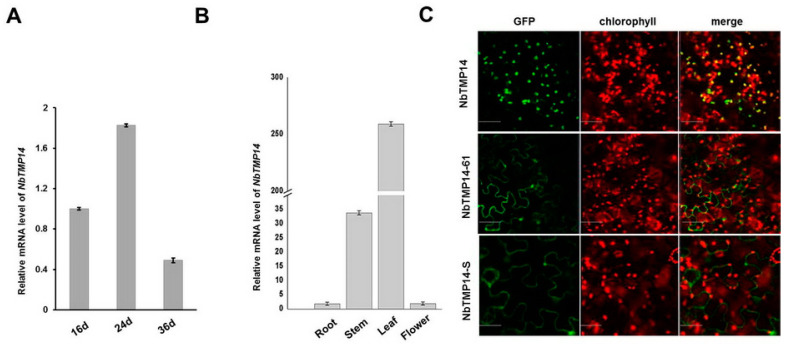
RT-qPCR assay of *NbTMP14* gene expression and subcellular localization of NbTMP14 in *N. benthamiana*. (**A**) NbTMP14 relative expression levels in different stages of wild-type *N. benthamiana*. Bars represent the mean ± standard error. (**B**) NbTMP14 relative expression levels in different organs of wild-type *N. benthamiana*. (**C**) Fluorescence of NbTMP14, NbTMP14-S (signal peptide), and NbTMP14–61 (mature protein) in leaf epidermal cells of *N.benthamiana*; chlorophyll autofluorescence was used as a marker. Scale bars represent 100 µm.

To analyze expression patterns of NbTMP14, qRT-PCR was performed using total RNA isolated from different *N. benthamiana* samples. The results showed that NbTMP14 expression reached a peak level at 24 days old (Figure 2A). NbTMP14 was mainly expressed in the leaf and stem tissue of *N. benthamiana*, having significantly higher expression in leaves, while the roots and flower had a very low quantity of the NbTMP14 transcript (Figure 2B).

To confirm chloroplast localization of NbTMP14, a green fluorescence protein (GFP) reporter was fused to the C-terminus of a signal peptide (NbTMP14-S-GFP), mature protein (NbTMP14-61-GFP), and the full length (NbTMP14-GFP) of NbTMP14, respectively, then transiently expressed in *N. benthamiana* leaves. NbTMP14-GFP signals overlapped nicely with chlorophyll autofluorescence, suggesting that NbTMP14 guided the GFP to enter the chloroplasts; however, neither the signal peptide nor mature protein of NbTMP14 could be located in the chloroplast (Figure 2C). The results indicated that the full sequence of NbTMP14 was essential for chloroplast localization of NbTMP14.

### 3.3. TSWV Infection Caused Structural Alteration of Chloroplasts by Disturbing NbTMP14 Expression and Subcellular Localization 

**Figure 3:**
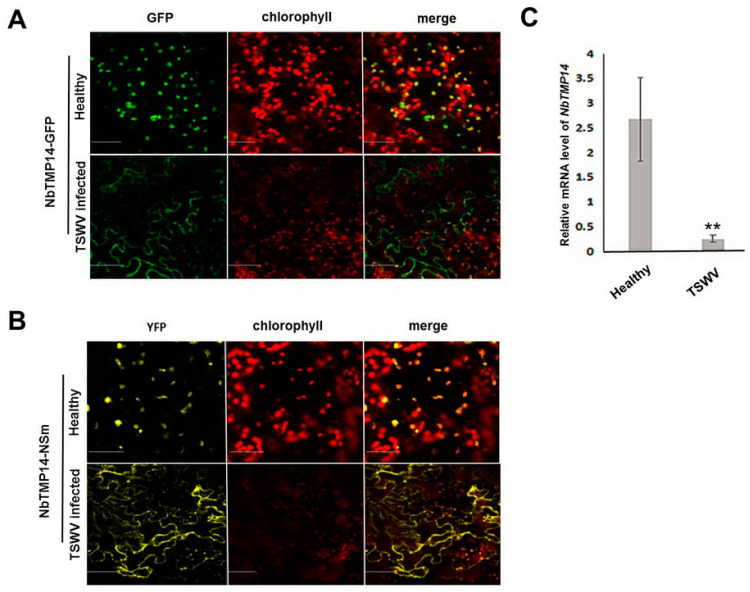
NbTMP14 expression and subcellular localization were disturbed by TSWV infection. (**A**) The subcellular localization of *NbTMP14* was compared between healthy and TSWV-infected plants at 10 dpi. Scale bars represent 100 µm. (**B**) The interaction site of NbTMP14/NSm was compared between healthy and TSWV-infected plants. Scale bars represent 100 µm. (**C**) The expression levels of *NbTMP14* were compared between healthy and TSWV-infected plants; *NbTMP14* was significantly downregulated after viral infection. Bars represent the mean ± standard error, ** indicates the significant differences (Student’s *t*-test, ** *p* < 0.01).

We speculated that the interaction of NSm and NbTMP14 during TSWV infection may affect expression of NbTMP14 and chloroplast morphology, thus resulting in viral symptom development. Results showed NbTMP14 could not be transported into chloroplasts in TSWV-infected cells and displayed a cell membrane localization pattern (Figure 3A). The interaction site of NbTMP14/NSm was also disrupted (Figure 3B), which indicated that the abnormal subcellular localization of NbTMP14 may lead to abnormal chloroplast development. The expression level of NbTMP14 was compared between TSWV-infected and healthy plants; the transcription of NbTMP14 was significantly downregulated during viral infection (Figure 3B).

**Figure 4:**
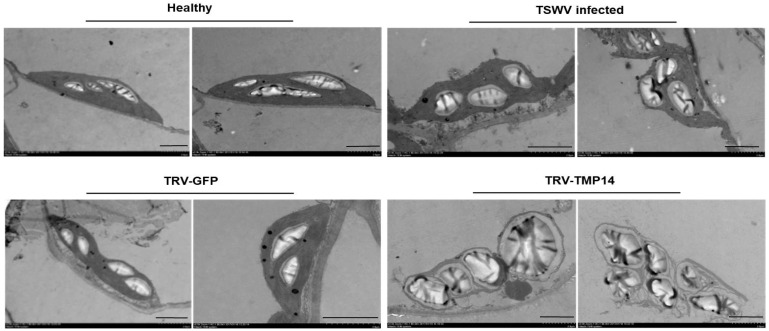
Transmission electron micrographs of *N. benthamiana* cells. The chloroplasts of TSWV-infected plants were abnormal and had larger starch granules. In the NbTMP14 knock-down plants, most chloroplasts were broken, and starch granules were enlarged. Scale bars represent 2 µm.

To verify if the structure of the chloroplast was damaged by viral infection, we prepared thin sections from the fixed embedded *N. benthamiana* leaf tissues of TSWV-infected, healthy, and NbTMP14 knock-down plants. Under the electron microscope, the chloroplasts of TSWV-infected plants were abnormal and had larger starch granules; in the NbTMP14 knock-down plants, most chloroplasts were broken, and starch granules were enlarged (Figure 4). 

These findings further confirmed that TSWV infection interferes with chloroplast development by disturbing NbTMP14 expression and subcellular localization.

### 3.4. NSm Interacted with Mature Proteins and Disturbed the Expression of NbTMP14

**Figure 5:**
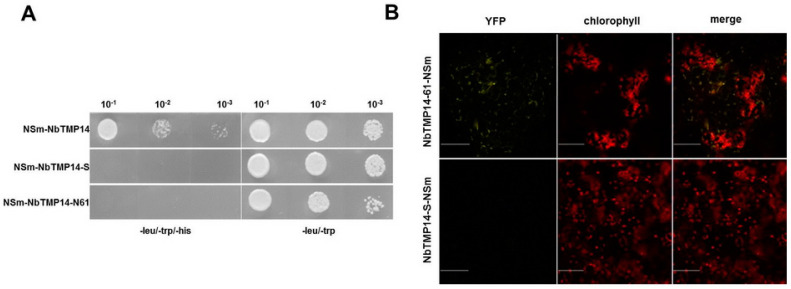
TSWV interacted with the mature form of the NbTMP14 protein. (**A**) Verification of TSWV interacting with the mature form of the NbTMP14 protein by yeast two-hybrid system. (**B**) The interaction between TSWV and the mature form of the NbTMP14 protein was investigated by BiFC in leaves of *N. benthamiana*. Scale bars represent 100 µm.

To analyze whether the interaction between TSWV NSm and NbTMP14 can be mapped either to the mature protein region or the signal peptide region, NbTMP14 mutants were generated in Y2H systems; the results indicated that TSWV NSm interacts with the mature protein of NbTMP14 (Figure 5A). 

**Figure 6:**
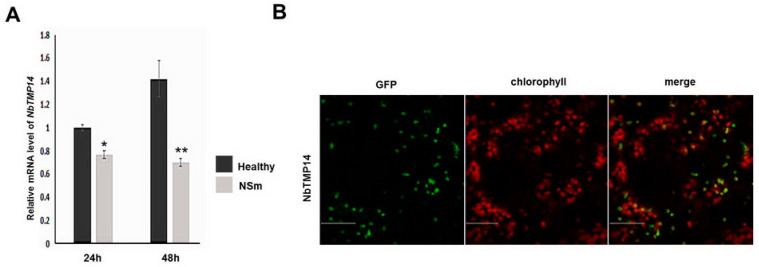
Transient expression of NSm downregulates NbTMP14 expression. (**A**) The expression of *NbTMP14* was compared between healthy and transient expression of NSm plants at 24 and 48 h; NbTMP14 expression was significantly reduced compared with the control sample. Bars represent the mean ± standard error, ** indicates the significant differences (Student’s *t*-test, * *p* < 0.05, ** *p* < 0.01). (**B**) The subcellular localization of NbTMP14 in transient expression of NSm plants was not influenced by NSm. Scale bars represent 100 µm.

These results were further confirmed in plant cells using a BiFC assay. Without the chloroplast-located signal peptide, the interacting protein could not be imported into the chloroplast of *N. benthamiana* cells (Figure 5B). To test if NSm was sufficient to induce downregulated expression and change the subcellular localization of NbTMP14, we transiently expressed NSm in *N. benthamiana* leaves. NbTMP14 expression was significantly reduced compared to the control sample (Figure 6A), while the subcellular localization of NbTMP14 was not influenced by NSm (Figure 6B).

### 3.5. NbTMP14 Played a Positive Role in Resistance to TSWV

**Figure 7:**
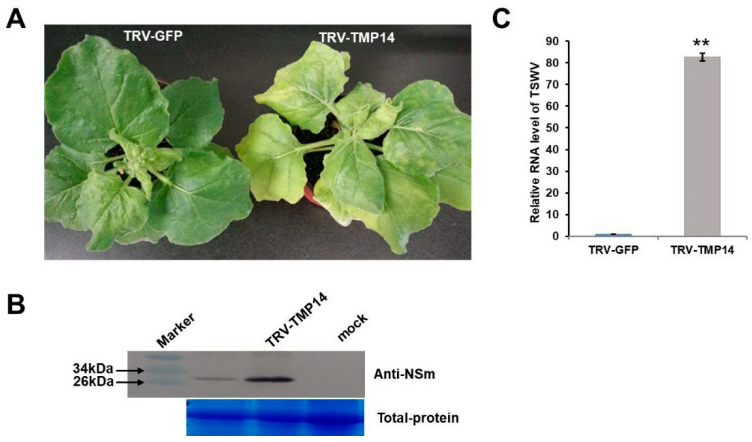
Accumulation of TSWV is upregulated in NbTMP14-silenced plants. (**A**) Symptoms of TRV2-GFP and TRV2-NbTMP14 plants after 15 days of TSWV infection. (**B**) Western blot detected the differing accumulation of TSWV between TRV2-GFP and TRV2-NbTMP14 plants. (**C**) RT-qPCR shows the accumulation of TSWV RNA between TRV2-GFP and TRV2-NbTMP14 plants. Bars represent the mean ± standard error, ** indicates significant differences (Student’s *t*-test, ** *p* < 0.01).

To elucidate the role of NbTMP14 in TSWV infection, the TRV VIGS system was employed to knock-down the transcript of *NbTMP14*. The silencing effects on TRV-TMP14 plants were confirmed by comparing their expression levels with TRV-GFP control plants (Figure S1). Silenced plants were infected with TSWV and monitored for symptom development, and virus accumulation was tested by the western blot assay. The results showed that the disease severity in the inoculated TMP14-silenced plants increased, and the silencing expression of NbTMP14 substantially increased TSWV accumulation in TRV-TMP14 compared with TRV-GFP plants (Figure 7).

**Figure 8:**
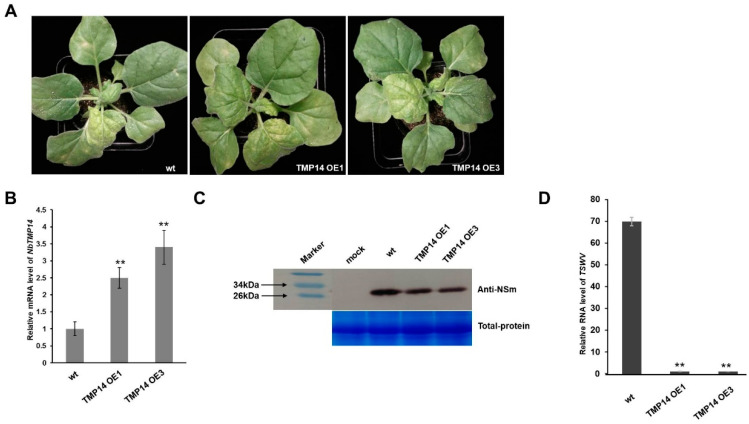
Enhanced TSWV resistance of *N. benthamiana* plants overexpressing *NbTMP14*. (**A**) Symptoms of the wild-type and NbTMP14 overexpression (OE) plants (the lines TMP14 OE1 and TMP14 OE3 were randomly selected) after 15 days of TSWV infection. (**B**) Expression levels of NbTMP14 in transgenic *N. benthamiana*. Bars represent the mean ± standard error, ** indicates the significant differences (Student’s *t*-test, ** *p* < 0.01). (**C**) Western blot detected the differing accumulation of TSWV between wild-type and NbTMP14 OE plants. (**D**) RT-qPCR shows the accumulation of TSWV RNA between the wild-type and NbTMP14 OE plants. Bars represent the mean ± standard error, ** indicates significant differences (Student’s *t*-test, ** *p* < 0.01).

To further determine the role of NbTMP14 in *N. benthamiana* responses to TWSV infection, we used *Agrobacterium*-mediated transformation to produce transgenic plants overexpressing the NbTMP14-GFP fusion protein driven by the CaMV 35S promoter as well as plants overexpressing the GFP protein as a control (Figure S2). The expression of NbTMP14 in transgenic plants was analyzed by RT-qPCR (Figure 8B), and then the plants were inoculated with TSWV. The NbTMP14 overexpression (OE) plants showed milder wilt symptoms compared to those of wild-type plants (Figure 8A), accompanied with decreased accumulation of TSWV (Figure 8C). The results of symptom development and virus detection indicated that the TSWV symptoms in *N. benthamiana* plants were negatively correlated with the accumulation levels of the NbTMP14 protein.

## 4. Discussion

In plants, chloroplasts are a common target of plant viruses for viral pathogenesis [37,38,39]. During viral infection, viruses not only disturb the host chloroplast’s normal function, they also change the chloroplast structure, which is associated with symptom development [27,40,41]. For example, *Tobacco mosaic virus* (TMV) coat protein (CP) localizes in the chloroplast and forms pseudovirions, in which chloroplast transcripts are encapsidated by TMV CPs, bind to the photosystem II (PS II) complex, and inhibit PS II activity [42,43]. *Cucumber mosaic virus* (CMV) CP also possesses the virulence to induce symptoms in CMV-infected tobacco plants, which is associated with chloroplast abnormalities [39,44]. In the present study, we identified a chloroplast protein, NbTMP14, which was targeted by TSWV NSm and revealed several mechanisms underlying TSWV infection and symptom development.

Previous studies have shown that NSm is a symptom determinant that can induce infection-like symptoms in transgenic plants [16,21,22,45]. In this study, we provided further evidence that there are larger starch grains in NbTMP14 silencing and TSWV-infected plants. This phenomenon was also observed in the mesophyll of tobacco expressing NSm protein, and was attributed to plasmodesmata closure [22]. Combining our results with previous reports, we conclude that NbTMP14 is one of the critical host factors targeted by TSWV NSm that interferes with metabolism of starch in chloroplasts. However, transient expression of NSm alone did not change NbTMP14 chloroplast localization based on our experiments. These results imply that other viral components may be involved in the interaction, and there is no direct interaction between NbTMP14 and other viral proteins (data not shown). The TSWV nucleocapsid N interacts with NSm, and we speculated that other viral component(s) help NSm, thereby trapping NbTMP14 in the cytoplasm.

Plant chloroplasts also play an important role in the defense response against viruses. The synthesis of plant hormones, such as salicylic acid (SA), jasmonic acid (JA) and abscisic acid (ABA), which regulate plant defense to viruses, greatly relies on chloroplastic machinery [46,47,48]. In addition, chloroplasts are major sources of the production of reactive oxygen species (ROS) [49,50,51,52]. Silencing of NbTMP14 gene expression disturbed chloroplast development and enhanced TSWV accumulation, suggesting that NbTMP14 could play an antiviral role and chloroplasts play an important defense role during TSWV infection. In addition to the silencing approach, we analyzed TSWV accumulation in NbTMP14 overexpression plants, in which viral protein accumulation was significantly reduced. These results further demonstrated that NbTMP14 may be integral to chloroplast-mediated defense machinery.

In summary, our findings provide the first evidence that the chloroplast thylakoid membrane protein NbTMP14 plays an important role in TSWV infection, and NSm interaction with NbTMP14 modulates TSWV symptoms through disruption of chloroplast structure and function. This report also indicates for the first time that TMP14 in plants is involved in viral symptom development.

## Data Availability

The data presented in this study are available in this article.

## Supplementary Materials

The following are available online at https://www.mdpi.com/1999-4915/13/3/427/s1, Figure S1: TRV-induced *NbTMP14* gene silencing resulted in a leaf yellowing phenotype, Figure S2: Two transgenic lines of *NbTMP14* overexpression plants, Table S1: The primers used in this study.
