# Supplementary material for: NbTMP14 Is Involved in Tomato Spotted Wilt Virus Infection and Symptom Development by Interaction with the Viral NSm Protein
Source: Viruses. 2021 Mar 7;13(3):427. doi: 10.3390/v13030427 (PMC7999277; doi:10.3390/v13030427)
Supplement: Supplementary file 1 [file viruses-13-00427-s001.zip › suppl/Fig S1.pdf]

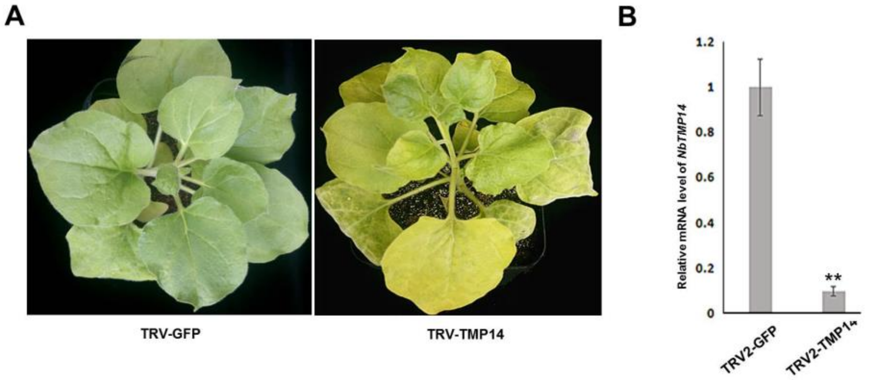

Fig S1. TRV-induced NbTMP14 gene silencing resulted in a leaf yellowing phenotype. A. The symptoms of TRV2-GFP and TRV2-NbTMP14 plants. B. The expression of *NbTMP14* was compared between TRV2-GFP and TRV2-NbTMP14 plant.
