# Supplementary material for: NbTMP14 Is Involved in Tomato Spotted Wilt Virus Infection and Symptom Development by Interaction with the Viral NSm Protein
Source: Viruses. 2021 Mar 7;13(3):427. doi: 10.3390/v13030427 (PMC7999277; doi:10.3390/v13030427)
Supplement: Supplementary file 1 [file viruses-13-00427-s001.zip › suppl/Table S1.docx]

| Experiment name | Primer name | Primer sequence(5’-3’) |
| --- | --- | --- |
| Clone *TMP14* | 5’TMP14 | ATGGCCTCAACTACCTCCCC |
|  | 3’TMP14 | TCAGCTGCTCCCAATTACTT |
| Clone *NSm* | 5’NSm | ATGTTGACTCTTTTCGGTAA |
|  | 3’NSm | TTATATTTCATCAAAAGACA |
| Yeast [Two-hybrid](D:/Youdao/Dict/7.5.2.0/resultui/dict/?keyword=two-hybrid)  pPR3-N  pBT3-STE | 5’TMP14 Sfi | AAGGCCATTACGGCCGCCTCAACTACCTCCCC |
|  | 3’TMP14 Sfi | TTGGCCGAGGCGGCCTCAGCTGCTCCCAATTACTT |
|  | 5’TMP14-S Sfi | AAGGCCATTACGGCCGCCTCAACTACCTCCCC |
|  | 3’TMP14-S Sfi | TTGGCCGAGGCGGCCGCCGTCGACCAAAGTCGATG |
|  | 5’TMP14-61 Sfi | AAGGCCATTACGGCCAAGACAACTCGTCAGTCAGC |
|  | 3’TMP14-61 Sfi | TTGGCCGAGGCGGCCTCAGCTGCTCCCAATTACTT |
|  | 5’NSm Sfi | AAGGCCATTACGGCCTTGACTCTTTTCGGTAATAA |
|  | 3’NSm Sfi | TTGGCCGAGGCGGCCCCTATTTCATCAAAAGACAACT |
| [Bimolecular Fluorescence Complementation](D:/Youdao/Dict/7.5.2.0/resultui/dict/javascript:;)  pYNE  pYCE | 5’NSm EcoR1 | CGGAATTCTTGACTCTTTTCGGTAATAA |
|  | 3’NSm Spe1 | GGACTAGTTATTTCATCAAAAGACAACT |
|  | 5’TMP14 EcoR1 | CGGAATTCGCCTCAACTACCTCCCC |
|  | 3’TMP14 Spe1 | GGACTAGTGCTGCTCCCAATTACTT |
|  | 5’TMP14-S EcoR1 | AAGGCCATTACGGCCTTGACTCTTTTCGGTAATAA |
|  | 3’TMP14-S Spe1 | GGACTAGTGCCGTCGACCAAAGTCGATG |
|  | 5’TMP14-61 EcoR1 | CGGAATTCAAGACAACTCGTCAGTCAGC |
|  | 3’TMP14-61 Spe1 | AAGGCCATTACGGCCTTGACTCTTTTCGGTAATAA |
| Subcellular Localization  p35S-GFP | 5’TMP14 BamH1 | CGGGATCCCGCCTCAACTACCTCCCC |
|  | 3’TMP14 Sal1 | GCGTCGACGCTGCTCCCAATTACTT |
| VIGS  pTRV_2_ | 5’TMP14 EcoR1 | CGGAATTCGCCTCAACTACCTCCCC |
|  | 3’TMP14 Sma1 | TCCCCCGGGGTTGCTATATCAGCTGATGA |
| NSm Overexpression | 5’NSm Sal1 | GCGTCGAC ATGTTGACTCTTTTCGGTAA |
|  | 3’NSm BamH1 | CGGGATCC TATTTCATCAAAAGACAACTG |
| TMP14-GFP Overexpression  pBI121 | 5’TMP14 In-Fusion | TCGCAGGATCCCCGGGTGGTCAGTCCCTTATGGCCTCAACTACCTCC |
|  | 3'TMP14 overlap | GCTCACCATGCTGCTCCCAATTACTTC |
|  | 5’eGFP overlap | GGGAGCAGCATGGTGAGCAAGGGCGA |
|  | 3’eGFP In-Fusion | GGGGAAATTCGAGCTCTTACTTGTACAGCTCGTCCATG |
| Real-time PCR | 5’TMP14 | GCAAGAAATGTGGTCGCAATG |
|  | 3’TMP14 | ACACCAGGAATCAGAGGAAGC |
|  | 5’NSm | CATTTGATGTGCAGCCAAGAATA |
|  | 3’NSm | TCTTTATCAGCTCTGGGTGAATC |
|  | 5’Actin | TGGCTTACATTGCTCTTGACTA |
|  | 3’Actin | ATGTTTCCGTACAGATCCTTTC |

Table S1. The primers used in this study
