# Supplementary figures and images for: NbTMP14 Is Involved in Tomato Spotted Wilt Virus Infection and Symptom Development by Interaction with the Viral NSm Protein
Source: Viruses. 2021 Mar 7;13(3):427. doi: 10.3390/v13030427 (PMC7999277; doi:10.3390/v13030427)

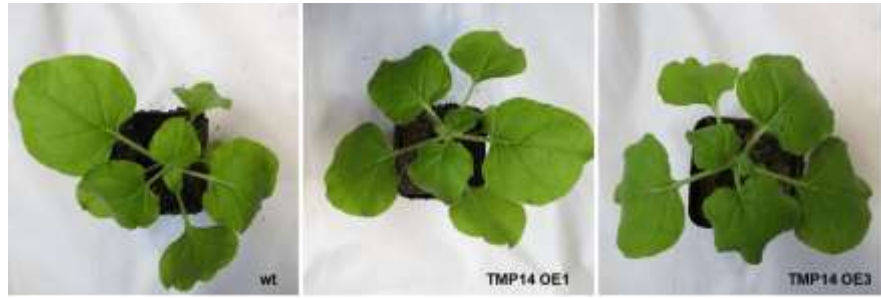

Figure S2. Two transgenic lines of NbTMP14 overexpression plants

Supplement: Supplementary file 1 [file viruses-13-00427-s001.zip › suppl/Fig S2.pdf]
